# Supplementary material for: Splice-Junction-Based Mapping of Alternative Isoforms in the Human Proteome
Source: Cell Rep. Author manuscript; Available in PMC 2020 Jan 15. (PMC6961840; doi:10.1016/j.celrep.2019.11.026)
Supplement: 3 [file NIHMS1546469-supplement-3.zip › DF2/PXD006675/AorticValve-12-Q9BX66-MSSAISPTPEISSETPGYIYSSNFHAVK.pdf]

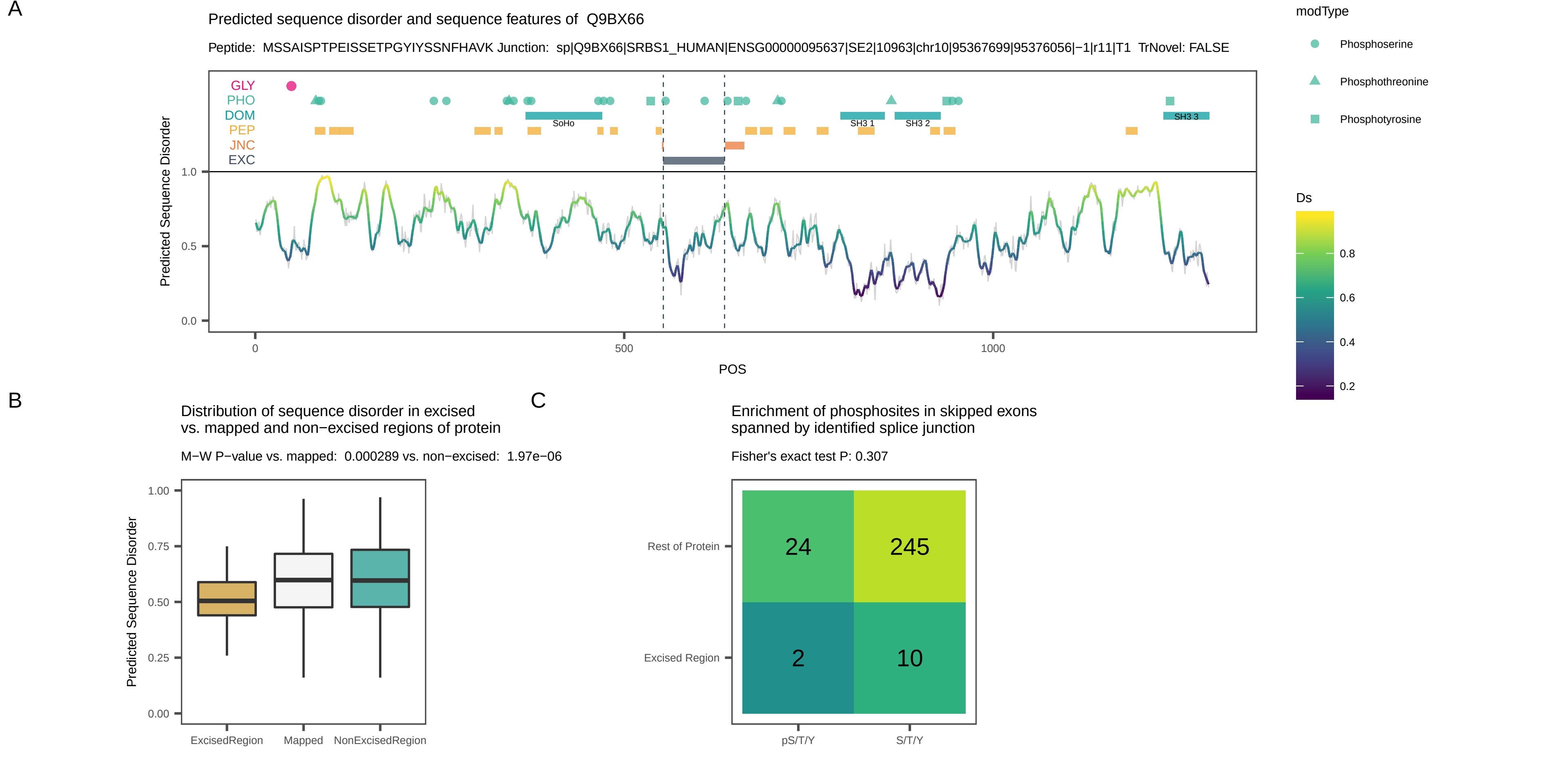

A
Predicted sequence disorder and sequence features of Q9BX66
Peptide: MSSAISPTPEISSETPGYIYSSNFHAVK Junction: sp|Q9BX66|SRBS1_HUMAN|ENSG00000095637|SE2|10963|chr10|95367699|95376056|−1|r11|T1 TrNovel: FALSE
GLY
PHO
DOM
PEP
JNC
EXC
SoHo
SH3 1
SH3 2
SH3 3
Predicted Sequence Disorder
1.0
0.5
0.0
0
500
1000
POS
modType
Phosphoserine
Phosphothreonine
Phosphotyrosine
Ds
0.8
0.6
0.4
0.2
B
Distribution of sequence disorder in excised
vs. mapped and non−excised regions of protein
M−W P−value vs. mapped: 0.000289 vs. non−excised: 1.97e−06
Predicted Sequence Disorder
1.00
0.75
0.50
0.25
0.00
ExcisedRegion
Mapped
NonExcisedRegion
C
Enrichment of phosphosites in skipped exons
spanned by identified splice junction
Fisher's exact test P: 0.307
Rest of Protein
Excised Region
24
245
2
10
pS/T/Y
S/T/Y
